# Social Factors Influence Behavior in the Novel Object Recognition Task in a Mouse Model of Down Syndrome

**DOI:** 10.3389/fnbeh.2021.772734

**Published:** 2021-11-05

**Authors:** Cesar Sierra, Ilario De Toma, Lorenzo Lo Cascio, Esteban Vegas, Mara Dierssen

**Affiliations:** ^1^Center for Genomic Regulation, The Barcelona Institute for Science and Technology, Barcelona, Spain; ^2^Neurosciences Research Program, Hospital del Mar Medical Research Institute, Barcelona, Spain; ^3^Department of Genetics, Microbiology and Statistics, Section of Statistics, Faculty of Biology, University of Barcelona, Barcelona, Spain; ^4^Department of Experimental and Health Sciences, University Pompeu Fabra, Barcelona, Spain; ^5^Biomedical Research Networking Center for Rare Diseases (CIBERER), Barcelona, Spain

**Keywords:** Down syndrome, Ts65Dn, novel object recognition test, environmental and social factors, experimental reproducibility, individual variability

## Abstract

The use of mouse models has revolutionized the field of Down syndrome (DS), increasing our knowledge about neuropathology and helping to propose new therapies for cognitive impairment. However, concerns about the reproducibility of results in mice and their translatability to humans have become a major issue, and controlling for moderators of behavior is essential. Social and environmental factors, the experience of the researcher, and the sex and strain of the animals can all have effects on behavior, and their impact on DS mouse models has not been explored. Here we analyzed the influence of a number of social and environmental factors, usually not taken into consideration, on the behavior of male and female wild-type and trisomic mice (the Ts65Dn model) in one of the most used tests for proving drug effects on memory, the novel object recognition (NOR) test. Using principal component analysis and correlation matrices, we show that the ratio of trisomic mice in the cage, the experience of the experimenter, and the timing of the test have a differential impact on male and female and on wild-type and trisomic behavior. We conclude that although the NOR test is quite robust and less susceptible to environmental influences than expected, to obtain useful results, the phenotype expression must be contrasted against the influences of social and environmental factors.

## Introduction

Behavioral testing is critical in rodent models of brain disorders, but there are numerous influences on behavioral measures, of which we are unaware. This leads to experimental variability from unknown sources reducing reproducibility and may be one of the reasons that preclinical studies in rodents are often not corroborated in humans. This is especially important for Down syndrome (DS), the most common genetic cause of intellectual disability. DS has been extensively modeled in mice (Herault et al., 2017). In the last decades, new murine models have been developed, and numerous studies have shown neuropathological alterations that contribute to the cognitive and behavioral phenotypes found in humans (Rueda et al., 2020). The Ts65Dn is the most used mouse model of DS, which is trisomic for the region of mouse chromosome 16 orthologous to human chromosome 21, spanning the region just proximal to the Gabpa/App gene cluster to Znf295 (Reeves et al., 1995). Ts65Dn has been reported to recapitulate many of the DS hallmarks, including impairment of memory and attention, and neurodevelopmental dysfunction (Escorihuela et al., 1995, 1998; Demas et al., 1998; Hunter et al., 2003). However, phenotypic variation is a characteristic of DS, but its causes are not completely established (Roper and Reeves, 2006; Dierssen, 2012). Some variation may be attributed to different laboratory environmental influences on genetically identical mice (Crabbe et al., 1999). However, even though specific guidelines, such as the ARRIVE or PREPARE guidelines, have addressed the design and conduct of *in vivo* animal experiments, and recently, the Trisomy 21 Research Society has adapted such guidelines to DS models (Roper et al., 2020), the use of thoroughly planned and well-reported protocols does not automatically guarantee the reproducibility of the study.

Our purpose is to shed light on some of the social and environmental factors, usually neglected or not considered in DS research, in the hope that new research will take these variables into account in study designs and reports. We focused on the impact of social and environmental factors on the novel object recognition (NOR) task (Ennaceur, 2010; Leger et al., 2013). The NOR is an efficient and flexible method for studying learning and memory in mice, widely used in DS mouse models studies, and testing potential therapies for DS (Arqué et al., 2008; Fernandez and Garner, 2008; Souchet et al., 2019; Alemany-González et al., 2020). It assesses intrinsically motivated learning in which animals spontaneously engage, and thus, there is no need for positive or negative reinforcement, which may complicate the interpretation of results, especially in DS mouse models, as they are less sensitive to pain (Martínez-Cué et al., 1999; Vaughan et al., 2017) and more sensitive to calorie restriction (Vaughan et al., 2017). The time needed to implement the assay is quite short compared with other tests as it can be completed over 3 days: habituation, training, and testing. This allows for higher throughput overall, which makes it ideal for drug testing. Additionally, there are a number of variations of the task so that, for example, the retention interval can be modified to examine short-term or long-term memory. Pharmacological intervention prior to or after training, or prior to recall, allows investigating acquisition, early or late consolidation, or recall. The NOR is also much less stressful than other tests such as the Morris water maze. This is important, as there is evidence that Ts65Dn mice exhibit heightened reactivity to a variety of stressors (Dierssen et al., 1997; Turner et al., 2001; Martínez-Cué et al., 2005), complicating the interpretation of performance in tasks that use stressors to motivate learning (Arqué et al., 2008). Moreover, secondary factors may influence the outcomes of the test, interacting with variables such as age or sex of mice, or with the genetic background (Stasko and Costa, 2004). For example, both thigmotaxis and hyperactivity have shown by Ts65Dn mice (Coussons-Read and Crnic, 1996; Costa et al., 1999) may affect the performance in the NOR (Herault et al., 2017).

There have been great efforts to standardize environmental conditions and laboratory practices (Richter et al., 2009), as this would enhance the reproducibility of results over time and across laboratories. However, most of the efforts to reduce the variability deriving from the environment have failed (Crabbe et al., 1999), and the influence of those factors on the behavioral readouts in DS mouse models is unknown. For example, environmental enrichment could lead to an excess of social and/or physical stimulation in male Ts65Dn mice (Martínez-Cué et al., 2002), which may affect cognition by disturbing the emotional and behavioral components of learning (Martínez-Cué et al., 2005) in a gender-dependent manner. Our hypothesis is that behavioral readouts may be influenced by differences in social and environmental conditions that can interact with genetic factors.

We here systematically explored whether usually not considered moderators of behavior may have an impact on the behavioral readouts of the widely-used NOR test, namely, the social environment, the experience of the experimenter, and circadian and ultradian variations. We also investigated how these extrinsic factors interact with sex-related differences in wild-type and trisomic mice.

## Materials and Methods

### Animals

Experimental mice were generated by crossing of Ts65Dn females to C57/6Ei × C3H/HeSnJ F1 hybrid (B6EiC3) males. The parental generation was obtained from the research colony at the Jackson Laboratory [B6EiC3Sn a/A-Ts(1716)65Dn/J; Stock No: 001924]. Genotypes of mice were authenticated by PCR assays on mouse tail samples with protocols from the sources of the mouse strains. Mice were housed in standard cages (156 mm × 369 mm × 132 mm), with food and water available *ad libitum*. The housing room was maintained on a 12:12 light cycle. Light intensity in the housing room was 400 lux. Sawdust and nesting materials in each cage were changed once a week, but never on the day before or the day of testing, to minimize the disruptive and stressful effect of cage cleaning on behavior.

All experiments followed the principle of the “Three Rs”: replacement, reduction, and refinement, according to Directive 63 / 2010 and its implementation in the Member States. Sample sizes were calculated by statistical power analysis aiming to capture moderate to high correlations in each group (*r* > 0.4) with a power of 75%.

### Novel Object Recognition Test

**FIGURE 1:**
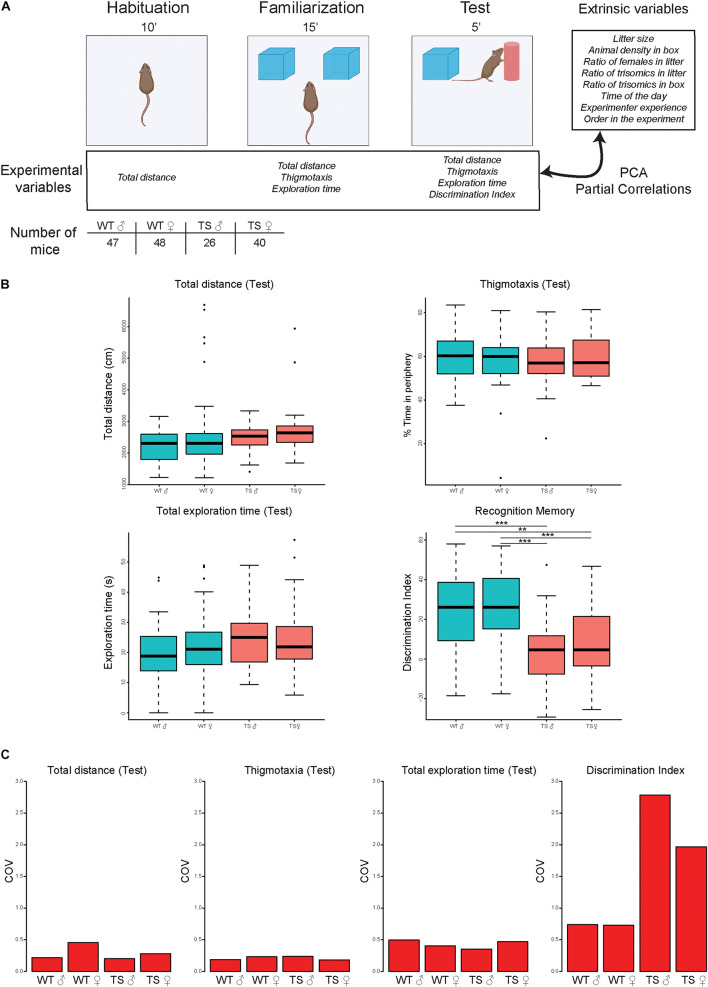
Novel object recognition performance in the test session in male and female wild-type and Ts65Dn mice. **(A)** Experimental schedule showing the different phases of the NOR [habituation (10 min); familiarization (15 min), and test (5 min)], indicating the experimental variables and extrinsic factors recorded (see section “Materials and methods”). The number of mice used in the experiment is depicted. WT, wild type; TS, Ts65Dn. **(B)** Box plots depicting the experimental variables (total distance traveled, percentage of time in the periphery, exploration time, and discrimination index) recorded in the test session for WT and TS mice. **p < 0.01; ***p < 0.001. **(C)** Coefficient of variation (COV) of the total distance traveled, thigmotaxis (percentage of time in the periphery), exploration time, and discrimination index across all groups of mice in the test session. WT, wild type male and female; TS, Ts65Dn male and female.

The novel object recognition (NOR) test is a relatively fast and efficient means for testing different phases of learning and memory in mice. The test was conducted in a square chamber (48 cm × 48 cm × 33 cm) made from black, non-porous plastic. Diffuse, low lighting was used with the center of the maze illuminated around 30 lux. We handled mice once a day for at least 1 min, 1 week prior to testing to reduce stress. In all cases, animals were picked up using the cupping method, as mice handled by tunnel and cupping methods show improved performance in behavioral tests compared with the traditional tail handling (Hurst and West, 2010). The NOR protocol consists of three phases (Figure 1A), namely, habituation, familiarization, and test sessions (Lueptow, 2017). During the habituation session, mice were allowed to freely explore the empty open field for 10 min; 24 h later, each mouse was returned to the arena containing two identical objects placed at symmetrical positions 5 cm from the arena wall and allowed to explore them freely for 15 min. During the familiarization session, most mice reached a minimum exploration total for both objects of 30 s. Mice not reaching this criterion at 15 min were excluded from the analysis, as it cannot be confirmed that they spent enough time exploring to learn/discriminate. After a retention interval of 24 h, the mouse was returned to the arena in which one of the objects was replaced by a novel object and let to explore both the familiar object and the novel object for a total of 5 min. The group sizes needed to achieve statistical significance with proper power tend to be high (around 20 mice/group). The number of animals used per group is reported in Figure 1A.

### Experimental Variables and Data Analysis

Figure 1A depicts the experimental design and variables considered. Locomotor activity was quantified as the total distance traveled in the apparatus during the experimental session. Thigmotaxis refers to the disposition to remain close to the walls of the apparatus. It is measured as the distance traveled or the percentage of time spent in the periphery of the apparatus. It decreases gradually during the first minutes of exploration and can be used as an index of anxiety (Simon et al., 1994). Exploration time is defined as the action of pointing the nose toward an object, at a maximum distance of 2 cm or touching it (Ennaceur and Delacour, 1988). In most studies, going around the objects or sitting on the object is not evaluated as exploration time. As such, the exploration time is only computed when the snout of the animal is directed toward the object, sniffing or touching it. Discrimination index (DI) is the most relevant parameter in the NOR, which is a measure of the recognition memory of the animal. It is calculated as the difference in exploration time for the familiar vs. the novel object, divided by the total amount of exploration, as shown by the following formula:


$$D\,I=\frac{T_{N\,o\,v\,e\,l}-T_{F\,a\,m\,i\,l\,i\,a\,r}}{T_{T\,o\,t\,a\,l}}$$

The DI is not influenced by the absolute value of the exploration times (Lueptow, 2017).

Supplementary Table 1 summarizes all the data recorded before and during the experiments. Extrinsic factors include social factors during the preweaning (size of the litter, number of trisomic mice, and number of females) and postweaning (animal density and number of trisomic mice in the cage), the experience of the experimenter (days from the beginning of the experiments), time of the day, and the order of a given animal in the experimental day. We analyzed their influence on experimental variables such as locomotor activity (total distance traveled) and thigmotaxis (percentage of time in the periphery) along with the habituation, familiarization, and test sessions (see Figure 1A).

### Statistical Analysis

To investigate statistical differences in experimental variables between groups, we conducted a one-factor ANOVA with four levels (WT males, WT females, TS males, and TS females) due to the unbalanced number of mice in one of the groups (TS males). For pairwise comparisons, the Shapiro–Wilk and Bartlett tests were used to assess the normality of values and the homogeneity of variances between groups, respectively. If the outcome was not normally distributed, the non-parametric Mann–Whitney–Wilcoxon, and Kruskal–Wallis tests were executed adjusting the *p*-value by Benjamini–Hochberg. This was the case in the following variables: thigmotaxis, discrimination index, total activity, and total object exploration time between genotypes or sex. All statistical analyses were two-tailed. *p*-values were considered to be significant when α < 0.05.

### Coefficient of Variation

We used the coefficient of variation (COV) as a standardized measure of the dispersion of a frequency distribution. It is defined as the ratio of the standard deviation σ to the mean μ and shows the extent of the variability in relation to the mean of the population.

### Principal Component Analysis

Principal component analysis (PCA) is the most commonly used technique to identify linear combinations of variables in a high-dimensional space best representing the variance that is present in the data. PCA identifies a linear combination of the original variables, called principal components, that accounts for the largest amount of the experimental variability. Once this first principal component is set, PCA finds successive orthogonal principal components that explain the maximum amount of the remaining variance. Finally, the original data and the original variables can be projected in this new space defined by the principal components. PCA was performed using the package FactoMineR. Missing values were imputed with the “imputePCA” function, estimating the number of components to use in the algorithm using cross-validation criteria implemented in the function “estim_ncpPCA.” The analysis was performed with the variables related to both the experiment itself and the condition before and during the experiment.

### Partial Correlations

A correlation matrix was used to investigate the dependence between different variables at the same time, with the partial correlation coefficient. This correlation measures the linear relationship between two variables, eliminating the effect of a set of other variables so it can avoid the risk of finding indirect relationships. This type of correlation measures the direct association between variables. The pcor function of the ppcor R package was used.

## Results

### Performance in the Novel Object Recognition Test

In the NOR paradigm, we detected slight hyperactivity in female Ts65Dn (TS) mice in the familiarization session that was not detected in the habituation or the test (Figure 1B, top left panel, Supplementary Table 2 and Supplementary Figure 1A). The time spent in the periphery of the apparatus (thigmotaxis) was not significantly different between genotypes (Figure 1B top right and Supplementary Figure 1B), nor between males and females. Regarding cognitive performance, the total time of exploration did not show genotype-dependent differences in the training (familiarization) (Supplementary Figure 1C) or test sessions (Figure 1B, bottom left panel). We detected impaired hippocampal memory, in both male and female TS mice as shown by a significantly reduced novel object discrimination (discrimination index; DI) compared with WT (*p* < 0.0001; Figure 1B bottom right panel).

### Coefficient of Variation

Interindividual variability in each group (WT men, WT women, TS men, and TS women) was calculated using the COV for the test session, as it includes the main parameter defining cognitive performance: the DI. Interindividual variation intragroup was very low for the total distance traveled, thigmotaxis, and exploration time. However, the DI had a high COV, especially in the trisomic groups, indicating that there is a high degree of variability in the memory performance of trisomic animals, with good and bad learners. Remarkably, the variation was higher in male compared with female TS mice (Figure 1C).

### Principal Component Analysis

**FIGURE 2:**
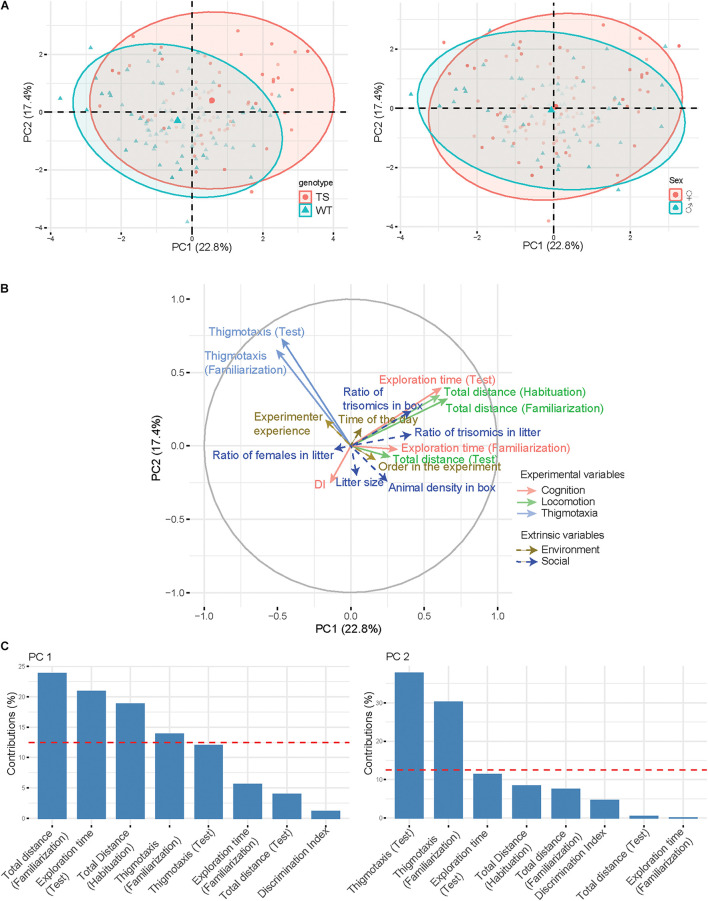
Principal component analysis. **(A)** PCA plotting the first two components (respectively, x-axis and y-axis). Points are plotted according to the genotype (left) or sex (right) and with the color intensity proportional to the importance of a principal component for a given observation (square cosine). Ellipses and their centroid are drawn for each group to show the direction of the separation on the axes. **(B)** Graph of variables along with the PCA plot. The variables used for the computation of the PCA are solid arrows, while supplementary variables are represented with dashed arrows. **(C)**. Barplot of variable contributions (loadings) for the first two principal components. A reference red dashed line is also shown on the barplot. This reference line corresponds to the expected value if the contribution were uniform.

The interindividual variability in the aforementioned experimental variables is represented in the PCA (Figure 2A). In this PCA, variables related to the locomotor activity, such as “total distance traveled along sessions,” and to cognition, such as “exploration time in the test session” are loaded on PC1 which accounted for 22.8% of the (between-individuals) variance (Figures 2B,C). High values of PC1 correspond to hyperactivity and increased exploration. In contrast, the second principal axis (PC2, 17.4% of variance) is dominated by the contribution of thigmotaxis and thus is mainly dependent on anxiety-like behavior. WT and trisomic animals are well separated in both dimensions of the PCA, showing a genotype effect on the NOR experimental variables. Instead, male and female mice show a similar distribution, indicating that sex does not influence behavior in this paradigm (Figure 2A).

We represented experimental variables (solid lines) in the two-dimensional space of the PCA (Figure 2B) in order to investigate how they interact and how they contribute to explaining the interindividual variability. Extrinsic (social and environmental) variables (dashed lines) were also represented in the same space. Interestingly, we detected a clear inverse relation between animal density in the box and thigmotaxis (animals that have a higher number of cage mates are less anxious and devote more time to object exploration during familiarization). The composition of the litter is also important, as the ratio of trisomic mice in the box seems to have a positive influence on the locomotor and exploratory activities of mice. Environmental factors do not have an overall impact on interindividual variability, suggesting that NOR is robust to factors such as the experience of the experimenter, the circadian rhythm, and the order of the animal in the experiment. These observations are similar when we take the same approach to investigate the intragroup variability and how the different variables interact inside of each group (Supplementary Figure 2). Particularly, the inverse relationship between the number of animals per cage and thigmotaxis is clearly maintained in all groups except for WT males. We also find that in all groups, social factors have generally a greater influence on behavior compared with environmental variables. Additionally, we also found other interesting group-specific observations such as a positive relationship between the ratio of trisomics in the litter and locomotor activity in WT males, as discussed below.

### Partial Correlations

**FIGURE 3:**
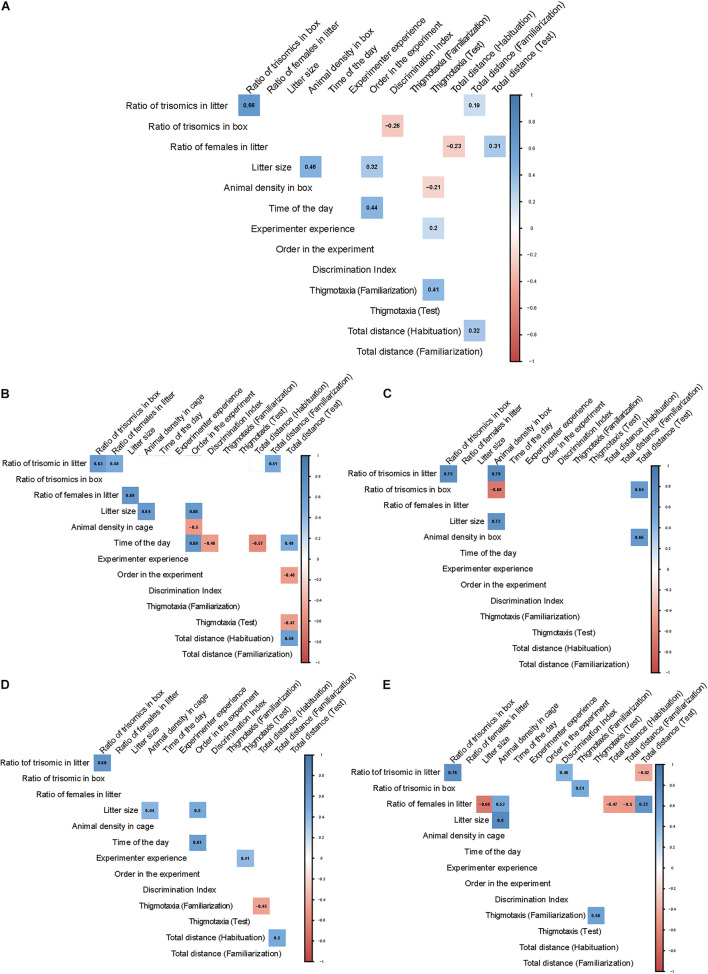
Correlograms showing partial correlations across the different groups of mice. Environmental factors and behavioral readouts are arranged in rows and columns. The correlation between variables is represented at their intersection with the correlation coefficient inside a colored square. The color of the square indicates if the correlation is positive (blue) or negative (red) and the color intensity, the weight of the correlation. The intersections in which the correlations did not reach significance (*p*-value > 0,05) were not colored. **(A)** Partial correlations corresponding to all mice tested. **(B)** WT males; **(C)** Trisomic male; **(D)** WT females; **(E)** Trisomic females.

In order to validate and quantify the one-to-one relation between experimental and extrinsic variables observed in the PCA, while controlling for the effects of the rest of the variables, we calculated the partial correlations between them in all groups. Figure 3 depicts the significant (*p* < 0.05) positive or negative partial correlations for all mice (Figure 3A) or by experimental group (Figures 3B–E). We also calculated the Pearson’s correlation *p*-values, which showed similar results and are depicted in Supplementary Figure 3.

When considering all the mice used (Figure 3A), similar to the PCA (Figure 2B) we mainly detected an influence of social factors on behavior, although the correlation coefficients were relatively low when all animals were included in the analysis. Specifically, the ratio of trisomic mice in the cage during the postweaning period negatively correlated with the DI (*r* = **−**0.26; *p* = 0.0041), as expected by the significant genotype-dependent differences in recognition memory. Also, the ratio of females in the cage during the preweaning period had a general influence on locomotor activity that is opposite in the habituation (*r* = **−**0.23; *p* = 0.012) and in the test session (*r* = 0.31; *p* = 0.0008). The total number of mice in the cage postweaning was negatively correlated with thigmotaxis in the test session (*r* = **−**0.21; *p* = 0.025), indicating that increased social influences reduce anxiety. Instead, the experience of the experimenter was correlated with thigmotaxis (*r* = 0.2; *p* = 0.02). As expected, mice that were more anxious in the familiarization session were also more anxious in the test session (*r* = 0.41; *p* < 0.0001), and those that were more active in the habituation retained this increased activity in the familiarization (*r* = 0.32; *p* = 0.0004).

When considering separately wild-type male mice (Figure 3B), we detected a negative correlation of the time at which the test was performed with the DI (*r* = **−**0.48; *p* = 0.02, Supplementary Figure 4A), suggesting that the later the test is performed, the worse male WT mice perform. Also, it influenced the locomotor activity that was negatively correlated in the habituation session (*r* = **−**0.57; *p* = 0.0006) and positively correlated in the familiarization (*r* = 0.43; *p* = 0.05) and in the test session (*r* = 0.49; *p* = 0.02). Interestingly, the ratio of trisomics in the cage before weaning had a positive correlation with the total distance traveled in the familiarization (*r* = 0,51; *p* = 0.018, Supplementary Figure 4B), indicating that litters with more trisomic mice in the first postnatal days lead to increased activity in some sessions of the NOR. We did not find any significant effect on the ratio of females in the cage before the weaning. Also, the number of mice in the cage had no impact on WT males neither in the preweaning period nor in the postweaning period. Finally, the experience of the experimenter had no impact on any of the experimental variables. The number of animals that underwent the test before also presented a negative correlation with locomotor activity in the test session.

Regarding trisomic males (Figure 3C), we only detected increased locomotor activity in the test session in cages with an increasing number of trisomic mice postweaning (*r* = 0.64; *p* = 0.04, Supplementary Figure 4D), which was also positively correlated with the total number of mice in the cage postweaning (*r* = 0.66; *p* = 0.03). No influences of any other factors were detected.

In wild-type females (Figure 3D), we could not detect any influence of social factors. Only the experience of the experimenter moderately correlated with thigmotaxis in the test session (*r* = 0.41; *p* = 0.03, Supplementary Figure 4C). Wild-type females more active in the habituation session were also more active in the test session (*r* = 0.38; *p* = 0.05). Finally, in trisomic females (Figure 3E), the ratio of trisomics in the preweaning period was positively correlated with the DI (*r* = 0.46; *p* = 0.02) of trisomic females in the test session and negatively correlated with the total distance traveled in the same session (*r* = **−**0.42; *p* = 0.05). Also, the ratio of trisomic mice in the postweaning period is positively correlated with thigmotaxis in the familiarization session (*r* = 0.51; *p* = 0.01, Supplementary Figure 4E). The ratio of females in the litter during the preweaning period correlates with locomotor activity in all sessions being negatively correlated in the habituation (*r* = **−**0.47; *p* = 0.02) and familiarization (*r* = **−**0.5; *p* = 0.01) but positively correlated in the test session (*r* = 0.72; *p* = 0.0001).

## Discussion

The Ts65Dn mouse model of DS recapitulates the hallmark hippocampus-dependent learning and memory dysfunction that characterizes the human disorder. Here we explored whether moderators of behavior usually not considered may have an impact on the behavioral readouts of the widely used NOR test, namely, the social environment, the experience of the experimenter, and circadian and ultradian variation. We also investigated how these extrinsic factors interact with sex-related differences in wild-type and trisomic mice.

The NOR, in its classical or modified versions, provides a unique opportunity to assess hippocampal-dependent recognition memory in a rapid, low-cost, and reliable manner. A large and growing number of studies also use the NOR to assess treatment efficacy in animal models of intellectual disability and more specifically in DS (Arqué et al., 2008; Fernandez and Garner, 2008; Souchet et al., 2019; Alemany-González et al., 2020). Because the novelty preference is spontaneous, no extensive training is required for either experimenters or mice, which makes the NOR test a widely accessible and time-efficient procedure. Despite these practical advantages, however, some recent observations have raised concerns about the validity of the NOR test DS mouse models, due to the high interindividual variability, whose sources are unknown. It can be affected by behavioral and/or physiological abnormalities characteristic of Ts65Dn mice (e.g., thigmotaxis and stress reactivity) and also by uncontrolled external factors, which complicate the interpretation of impaired performance.

Our NOR experimental design was validated as WT animals acquired memory after a 15 min familiarization training trial of unsupervised exposure to the cues. As anticipated, the WT mice had a pronounced preference for the novel object in the test session conducted 24 h after a single familiarization. Ts65Dn failed to display object recognition, showing equivalent time spent exploring the novel and familiar object during the test session. As a consequence, the DI showed a significantly reduced value both in trisomic male and female mice, while no sex-dependent differences were detected in either genotype.

Our results suggest that the recognition memory problem found in the Ts65Dn animals could not be attributed to a failure in cue sampling, since both genotypes spent the same total amount of time exploring the objects during the familiarization session. In fact, the amount of time mice spent investigating objects during the familiarization phase did not predict the magnitude of their novel-object preference, as also previously shown in rats (Gaskin et al., 2010; Gervais et al., 2016). One implication is that differences in the magnitude of a novelty preference should not be uncritically taken to reflect differences in memory ability.

Regarding locomotor activity, we detected the already known hyperactive phenotype in Ts65Dn male and female mice. Interestingly, opposite to what is generally believed, interindividual variation was very low for locomotor activity, thigmotaxis, or exploration time, not being qualitatively different across sex or genotype. Instead, DI showed the highest coefficient of variation, meaning that there was a higher degree of variability in cognitive results between individuals in the same group. The variation was much higher in trisomic mice, and of those, the highest was detected in trisomic males, contrary to the general assumption that females are more variable (Beery, 2018).

In our PCA, variables related to the locomotor activity, such as the total distance traveled, and to cognition, such as the exploration time, are loaded on PC1 which accounted for 22.8% of the (between-individuals) variance. High values of PC1 corresponded to hyperactivity and increased exploration. In contrast, the second principal axis (PC2, 17.4% of variance) was contributed by thigmotaxis, mainly dependent on anxiety-like behavior. WT and trisomic animals are well separated in both dimensions of the PCA, showing a genotype effect on the NOR. Instead, male and female mice show a similar distribution, indicating that sex does not influence behavior in this paradigm. Extrinsic (social and environmental) variables represented in the same space showed an inverse relation between animal density in the box and thigmotaxis, suggesting that animals that have a higher number of cage mates are less anxious and devote more time to object exploration during familiarization. Some studies have investigated the effects of animal density on a range of indicators of the ability to cope, including hormone activation, immune and biological functions and behavior (Christian, 1955; Brain and Nowell, 1970). Animals housed in pairs or in small groups showed the highest adrenocortical activity and a more pronounced decline in gonadal activity compared with all the other group sizes. This observation was explained as the subordinate mouse subjected to all social stress, while in larger groups this stress is distributed between a larger group of subordinate animals (Brain and Nowell, 1970). This would be especially relevant in trisomic mice, as they have been described as the main subdominant (Martínez-Cué et al., 2005), thereby increasing thigmotaxis, reducing their natural exploratory behavior, and affecting the performance of the NOR. The composition of the litter is also important, as the ratio of trisomics in the box seems to have a positive influence on the locomotor and exploratory activities of mice. Early social environments determine the kind of relationship and affiliative bonding among littermates and have been found to cause selective changes in different neural regulatory mechanisms that serve important behavioral functions (Laviola and Terranova, 1998).

These observations are similar when we take the same approach to investigate the intragroup variability and how the different variables interact inside of each group. Particularly, the inverse relationship between the number of animals per cage and thigmotaxis is clearly maintained in all groups except for WT males. We also find that in all groups, social factors have generally a greater influence on behavior compared with environmental variables. In fact, environmental factors do not have an overall impact on interindividual variability, suggesting that NOR is robust to factors such as the experience of the experimenter, the circadian rhythm, and the order of the animal in the experiment.

The correlation matrices reinforced the findings of the PCA. When analyzing all mice together, we detected a general influence on locomotor activity of the ratio of females in the cage during the preweaning period. Also, the total number of mice in the cage postweaning was negatively correlated with thigmotaxis, indicating that increased social influences reduce anxiety. However, we only detected increased locomotor activity in trisomic mice housed in cages with an increasing number of mice and also in those housed with an increased number of trisomic mice in the cage postweaning.

We would also have anticipated that litter size in the preweaning period would have affected trisomic mice more intensely, as it has been reported that there is a disproportionate loss of trisomic offspring in late gestation that continues through birth to weaning (Roper et al., 2006), suggesting reduced maternal care in trisomic dams. Early life factors (for example, level of maternal care received) may have long-lasting effects on brain functioning and abilities to cope with stress and therefore may also affect anxiety-like behavior in the offspring (Bechard et al., 2012). However, we did not detect any influence of the litter size on recognition memory or on thigmotaxis of any experimental group. Remarkably, the DI is not related to the exploration time during the familiarization. This suggests that a higher amount of time spent exploring the objects during the familiarization does not necessarily improve the ability to discriminate the objects during the test.

Interestingly, the group of trisomic females was the only group in which the discrimination index seems to be significantly influenced by specific social factors so that the ratio of trisomic in the preweaning period was positively correlated with the DI and negatively with the total distance traveled in the same session. However, in all other groups, the DI is not significantly correlated with social factors, indicating that the discrimination index is robust, and in most experimental groups, it is not influenced by social factors. Finally, the experience of the experimenter was negatively correlated with thigmotaxis so that the more experience the experimenter had, the less anxious were the trisomic females, although the opposite was detected for female wild-type mice.

## Conclusion

Our work indicates that social factors, such as the litter composition, the ratio of trisomics, or animal density in the home cage, differentially affect the phenotype expression in trisomic and wild-type mice. Specifically, our work warns that when using female trisomic mice, the number of trisomic mice in the preweaning period has to be considered when interpreting the results of the NOR, as it has a positive influence on the DI. The experience of the researcher and the time of the day in which the testing is performed may also affect the performance although only in specific experimental groups, and it does not influence recognition memory. Also of importance is the fact that increased animal density in the cage (up to four mice) reduced anxiety-related behavior, such as thigmotaxis, and increases exploration. Finally, it is worth mentioning that the intra-group variability was very low for variables such as activity (distance traveled), thigmotaxis, and exploration, but it was very high for the DI only in trisomic mice, being the highest in trisomic males. This recapitulates the situation in individuals with DS, in which the intelligence quotient can vary quite importantly across individuals ranging from severe to mild-moderate intellectual disability. We show that accurate recording, capture, and characterization of social and environmental parameters are key to understanding how they affect phenotypic expression and have a differential impact on trisomic and wild-type genotypes. Initiatives, such as the European Mouse Disease Clinic (EUMODIC), the International Mouse Phenotyping Consortium (IPMC), and recently, the Trisomy 21 Research Society have made important efforts to standardize assays and capture environmental metadata for phenotype assays.

## Data Availability Statement

The original contributions presented in the study are included in the article/Supplementary Material, further inquiries can be directed to the corresponding author/s.

## Ethics Statement

The animal study was reviewed and approved by the Ethics Committee of Parc de Recerca Biomèdica [Comité Ético de Experimentación Animal del PRBB (CEEA-PRBB)].

## Author Contributions

CS and MD contributed to the conceptualization and investigation. CS, MD, EV, and ID contributed to the methodology and visualization. ID contributed to the software. EV, ID, and CS contributed to the validation. EV and ID contributed to the formal analysis. MD contributed to the resources, supervision, and funding acquisition. LC and CS contributed to the data curation. MD, LC, and CS contributed to the writing (original draft preparation). MD and CS contributed to the writing (review and editing). All authors have read and agreed to the published version of the manuscript.

## Author Disclaimer

The views expressed in this article are those of the authors, and the European Commission is not responsible for any use that may be made of the information it contains.

## Conflict of Interest

The authors declare that the research was conducted in the absence of any commercial or financial relationships that could be construed as a potential conflict of interest.

## Publisher’s Note

All claims expressed in this article are solely those of the authors and do not necessarily represent those of their affiliated organizations, or those of the publisher, the editors and the reviewers. Any product that may be evaluated in this article, or claim that may be made by its manufacturer, is not guaranteed or endorsed by the publisher.
