# Supplementary material for: Social Factors Influence Behavior in the Novel Object Recognition Task in a Mouse Model of Down Syndrome
Source: Front Behav Neurosci. 2021 Nov 5;15:772734. doi: 10.3389/fnbeh.2021.772734 (PMC8602686; doi:10.3389/fnbeh.2021.772734)
Supplement: Supplementary file 1 [file Data_Sheet_1.pdf]

Supplementary Table 1. Extrinsic factors and experimental variables

**Social and circadian-rhythm-related variables**	**Experimental variables**
Litter size	Total distance habituation (Locomotor activity in habituation)
Ratio of females in the litter	Distance in periphery during habituation (Thigmotaxis in habituation)
Reatio of trisomics in the litter	Total distance familiarization (Locomotor activity in familiarization)
Animal density in the box	Percentage of time in periphery during familiarization (Thigmotaxis in familiarization)
Ratio of trisomics in the box	Distance in periphery during familiarization (Thigmotaxis in familiarization)
Time of the day	Exploration time during familiarization
Order of the animal in the experiment	Total distance test (Locomotor activity in test)
Experimenter's experience	Percentage of time in periphery during test (Thigmotaxis in test)
	Distance in periphery during test (Thigmotaxis in familiarization)
	Exploration time during test
	Discrimination index

Supplementary Table 2. Statistical significance p-values of the NOR variables during the habituation and familiarization sessions.

	ANOVA / Kruskal-Wallis	WT.f vs TS.f	WT.f vs WT.m	WT.f vs TS.m	TS.f vs WT.m	TS.f vs TS.m	WT.m vs TS.m
Total distance (Habituation) *	0.095	0.463	0.518	0.085	0.488	0.488	0.124
Total distance (Familiarization)	0.006	0.043	1.000	1.000	0.008	1.000	0.383
Thigmotaxis (Familiarization)	0.151	1.000	1.000	0.500	1.000	0.150	1.000
Exploration time (Familiarization) *	0.066	0.856	0.057	0.639	0.111	0.645	0.331

* denotes variables that do not meet homoscedasticity or normality.

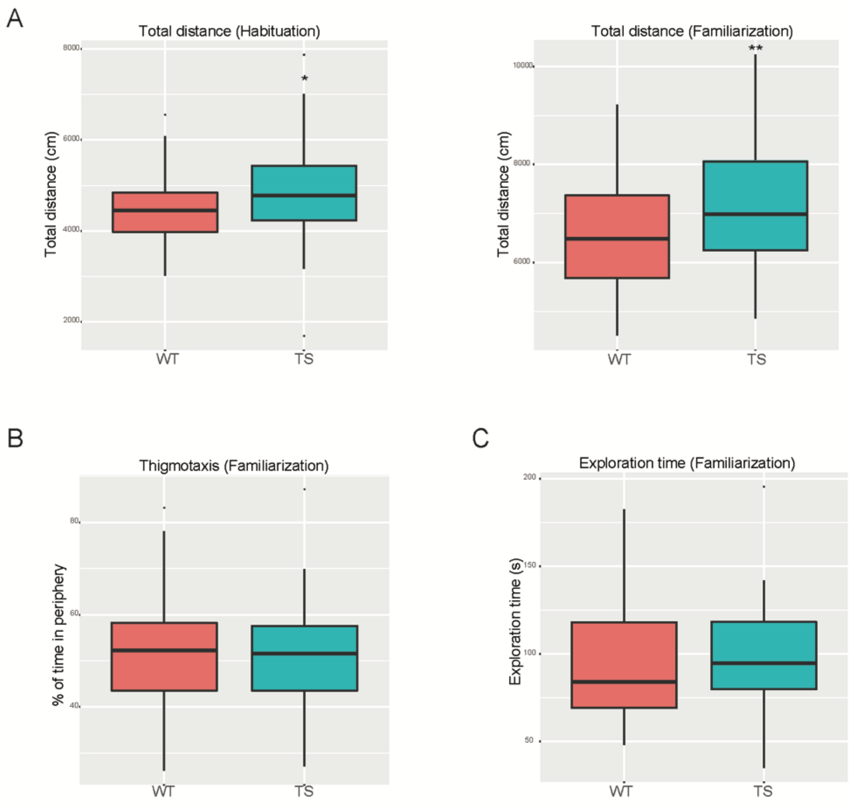

Supplementary Figure 1. Novel object recognition performance in the habituation and familiarization sessions in male and female wild type and Ts65Dn mice. Boxplots depicting the experimental variables: **A.** Total distance travelled in the habitation and familiarization sessions; **B.** Percentage of time in the periphery in the familiarization session, and **C.** exploration time recorded in the familiarization session for WT and TS mice. * $p< 0.05$; ** $p < 0.01$..

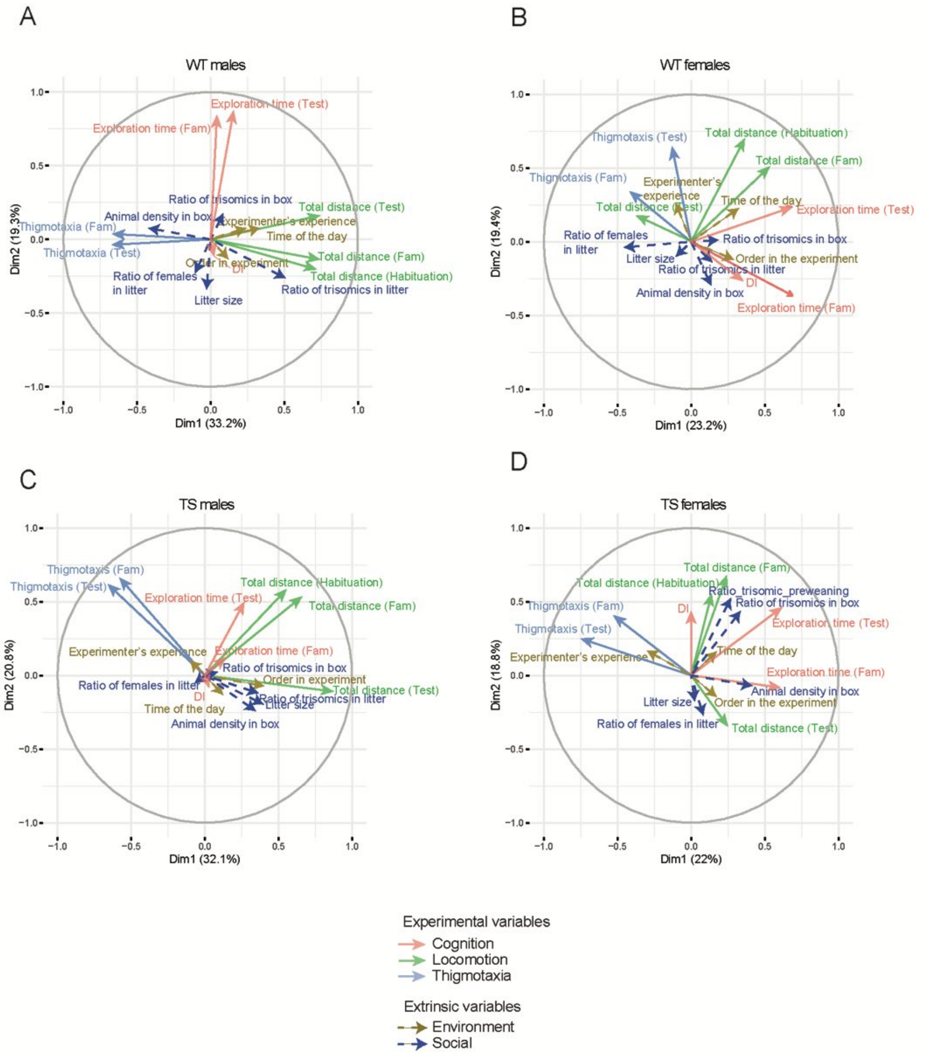

Supplementary Figure 2. Principal component analysis for each experimental group. Graph of variables along the PCA plot for **A.** WT males; **B.** WT females; **C.** TS males; **D.** TS females. The variables used for computation of the PCA are solid arrows, while supplementary variables are represented with dashed arrows.

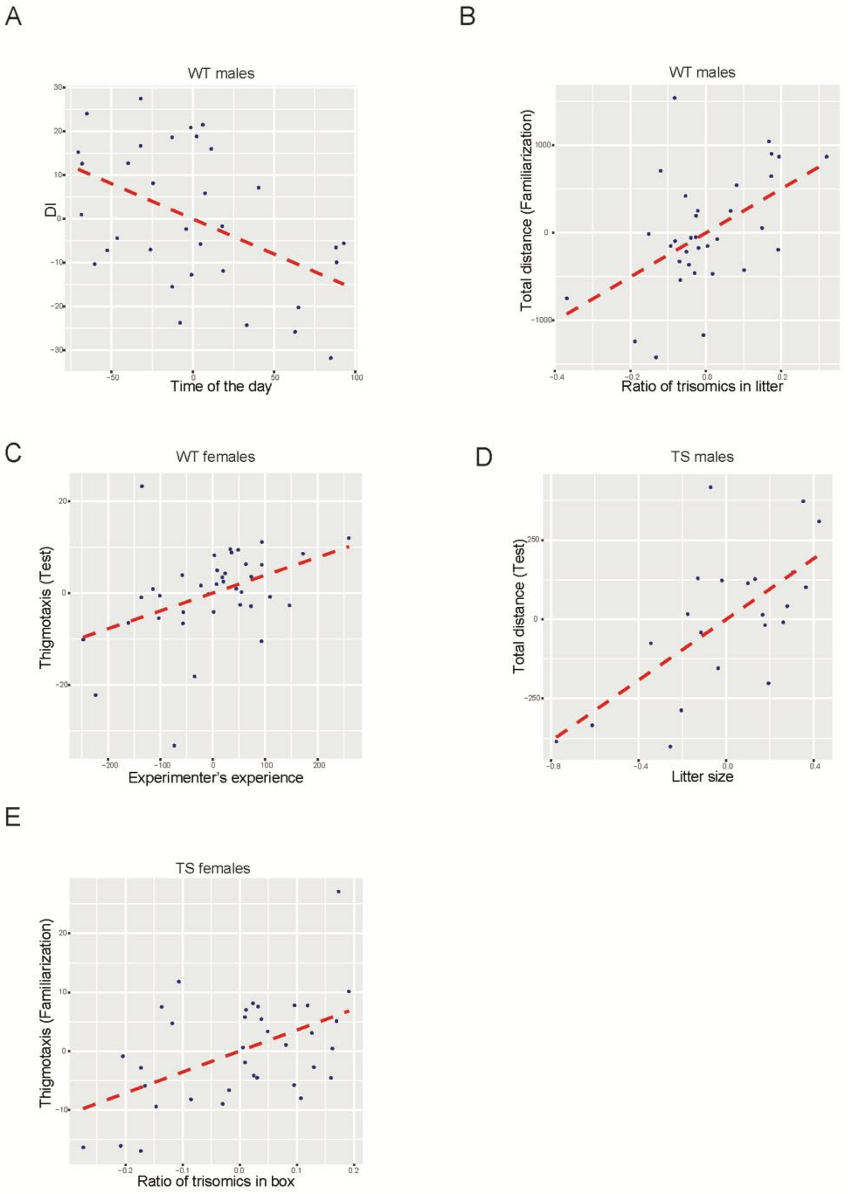

Supplementary Figure 3. Scatter plots of the most representative correlations. A. Negative correlation of the time at which the test was performed with the DI (r = -0.48; p = 0.02) in WT males. B. Positive correlation of the ratio of trisomics in the cage before weaning with the total distance travelled in the familiarization by WT male mice (r = 0,51; p = 0.018). C. Positive correlation of the experimenter's experience with thigmotaxis displayed by WT female mice in the test session (r = 0.41; p = 0.03). D. Positive correlation between locomotor activity displayed by trisomic males in the test session with an increasing number of trisomic mice postweaning (r = 0.64; p = 0.04). E. Positive correlation between the ratio of trisomic mice in the postweaning period with thigmotaxis displayed by female trisomic mice in the familiarization session (r = 0.51; p = 0.01,

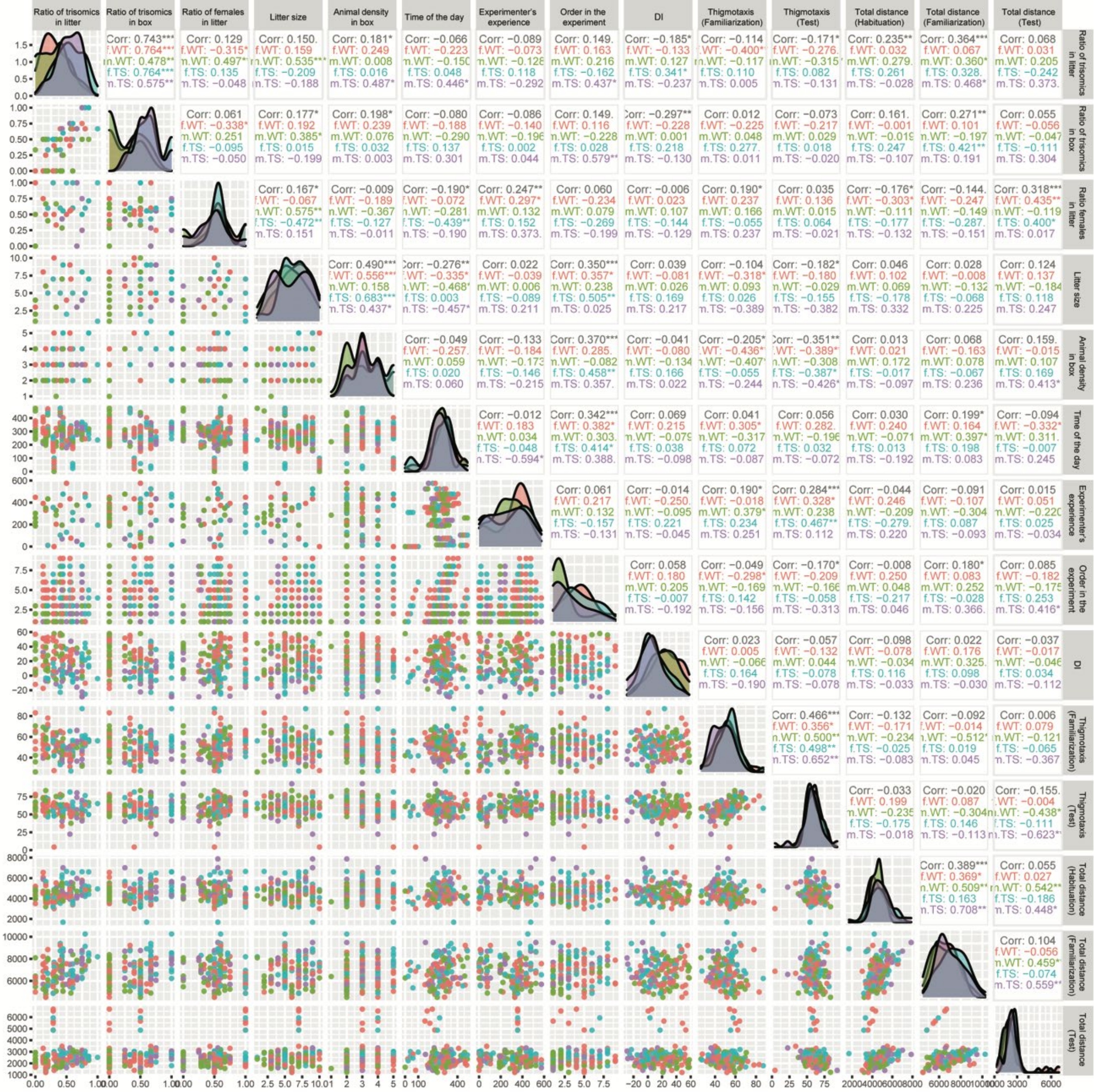

Supplementary Figure 4. Multivariate correlation matrix with grouping. The graph plots three pieces of information: i/ the correlation matrix between variables grouped by whether mice are WT male (green) WT female (red), trisomic male (purple) or trisomic female (light blue); ii/ the plot of the distribution of each variable by group; and iii/ the scatter plot with the trend by group. Numbers represent the value of r. *p < 0.05; ** p < 0.01; *** p < 0.001; Pearson correlation.
